# NLRP3 inflammasome activation in astrocytes restricts SARS-CoV-2 through gasdermin-D-driven IL-1β release

**DOI:** 10.3389/fimmu.2025.1703765

**Published:** 2026-01-20

**Authors:** Ingrid S. de Farias, Márcia Duarte-Barbosa, Natalia Salazar, Robert Andreata-Santos, Victoria Weise L. de Lucena, Juliana T. Maricato, Ricardo T. Gazzinelli, Luiz Mário Ramos Janini, Karina Ramalho Bortoluci

**Affiliations:** 1 Departamento de Farmacologia, Escola Paulista de Medicina/Universidade Federal de São Paulo (EPM/UNIFESP), São Paulo, SP, Brazil; 2 Programa de pós-graduação em Imunologia, Instituto de Ciências Biomédicas, Universidade de São Paulo (ICB/USP), São Paulo, SP, Brazil; 3 Departamento de Medicina, Escola Paulista de Medicina/Universidade Federal de São Paulo (EPM/UNIFESP), São Paulo, SP, Brazil; 4 Centro de Tecnologia em Vacinas, Universidade Federal de Minas Gerais, Belo Horizonte, Brazil; 5 Departamento de Microbiologia, Imunologia, Parasitologia, Escola Paulista de Medicina/Universidade Federal de São Paulo (EPM/UNIFESP), São Paulo, SP, Brazil

**Keywords:** astrocytes, gasdermin-D, IL-1β, inflammasome, NLRP3, SARS-CoV-2

## Abstract

Severe acute respiratory syndrome coronavirus 2 (SARS-CoV-2) is the third highly pathogenic coronavirus to emerge in humans in recent decades. Although primarily a respiratory virus, SARS-CoV-2 can invade the central nervous system (CNS), leading to severe neurological manifestations such as stroke, encephalopathy, and memory loss. However, the mechanisms by which neural cells control SARS-CoV-2 infection remain poorly understood. Here, we demonstrate that SARS-CoV-2 and its Nucleocapsid (N) and Spike (S) proteins induce classical NLRP3 inflammasome activation in astrocytes. Notably, astrocytes lacking NLRP3 or caspase-1 exhibit higher viral loads, indicating a crucial role of the NLRP3 inflammasome in astrocyte-mediated viral control. Similarly, gasdermin-D (GSDMD)-deficient astrocytes display increased susceptibility to infection, although their LDH release remains unaffected, suggesting that pyroptosis is not required for viral restriction. Instead, GSDMD deficiency leads to markedly reduced IL-1β secretion, and exogenous IL-1β rescues the impaired antiviral response in NLRP3-, caspase-1-, and GSDMD-deficient astrocytes. Our findings reveal that astrocytes autonomously control SARS-CoV-2 infection via the NLRP3-GSDMD-IL-1β axis, underscoring their active role in the neuroimmune response to viral infection.

## 1 Introduction

Severe acute respiratory syndrome coronavirus 2 (SARS-CoV-2) is the third highly pathogenic coronavirus to be identified in humans in the past two decades (1). Following its rapid global spread, the World Health Organization (WHO) declared COVID-19 a pandemic in March 2020. By September 1, 2023, approximately 694 million people worldwide had been infected with SARS-CoV-2, with an estimated 55–60% of cases presenting clinical symptoms of COVID-19 (2, 3). Although primarily a respiratory virus, SARS-CoV-2 can reach the central nervous system (CNS) through mechanisms that remain incompletely understood (4–6). Increasing evidence suggests that the virus induces neurological manifestations in a significant proportion of COVID-19 patients, including stroke, encephalopathy, and cognitive impairments (7–9). Human astrocytes have been identified as key sites for SARS-CoV-2 replication in the brain (10–14). Studies using cortical organoids and post-mortem brain tissues have shown that astrocytes are highly susceptible to infection and viral replication, even in the absence of significant ACE2 expression, suggesting the existence of alternative viral entry pathways (11).

Astrocytes, the most abundant glial cells in the CNS, play a fundamental role in maintaining brain homeostasis (15). They provide metabolic support to neurons, regulate neurotransmitter recycling, and maintain the integrity of the blood-brain barrier (16). Additionally, astrocytes express multiple Pattern Recognition Receptors (PRRs), allowing them to sense and respond to infections. However, the mechanisms underlying their autonomous antiviral responses, particularly in the context of SARS-CoV-2, remain poorly understood.

Among PRRs, inflammasomes have emerged as central components of the innate immune response to SARS-CoV-2 (17–27). The virus can infect monocytes and activate inflammasomes such as NLRP3 and AIM2. Overactivation of these platforms leads to pyroptosis and increased disease severity in individuals with elevated expression of inflammasome-related genes (e.g., *GSDMD, NLRP3, NLRC4*) (26). However, the role of inflammasomes in controlling SARS-CoV-2 spread remains unexplored, especially in the CNS.

Post-mortem studies have detected SARS-CoV-2 nucleocapsid protein in neurons, astrocytes, oligodendrocytes, and microglia, along with ACE2 expression and NLRP3 inflammasome components (28), and experimental data indicate that the viral Spike protein can activate NLRP3 in microglia via ACE2 and NF-κB signaling (29). However, whether astrocyte-intrinsic activation of inflammasomes contributes to antiviral defense during SARS-CoV-2 infection remains largely unexplored. This gap is critical, given that astrocytes not only represent major replication sites for the virus (30) but also influence the immune microenvironment of the CNS.

Astrocytes deficient in NLRP3 and caspase-1 exhibit increased susceptibility to infection, highlighting the crucial role of these molecules in regulating viral replication. Additionally, Gasdermin-D (*Gsdmd*)-deficient astrocytes exhibit higher viral loads and reduced IL-1β secretion, suggesting that IL-1β release is crucial for astrocyte-mediated viral containment. Notably, treatment with recombinant IL-1β rescues the antiviral response in astrocytes deficient in inflammasomes. Together, these findings reveal that astrocytes possess an intrinsic capacity to restrict SARS-CoV-2 replication through an inflammasome-dependent mechanism, highlighting their active role in the neuroimmune response to viral infection.

## 2 Materials and methods

### 2.1 Animals

This work was submitted and accepted by the Ethics Committee on Animal Use of the Federal University of São Paulo (UNIFESP) and is registered under number 1368130918. C57BL/6 (Wild-type), *Nlrp3^-/-^, Nlrc4^-/-^, Caspase-1/11^-/-^* mice (kindly provided by Dr. Richard Flavell, Yale University, USA) and *Gsdmd^-/-^* (kindly provided by Dr. Petr Broz, University of Lausanne and supplied by Prof. Sergio Costa Oliveira – University of São Paulo) were purchased from the Center for the Development of Experimental Models for Medicine and Biology (CEDEME) UNIFESP. All animals were maintained under specific pathogen-free (SPF) conditions in microisolators, with free access to water and food.

### 2.2 Primary cell culture of astrocytes

Primary microglia and astrocytes were obtained according to a previously published protocol (31). Briefly, postnatal day 0–3 (P0–P3) mice were used. Animals were anesthetized by inhalation of isoflurane (Cristália) at 3 - 4% in oxygen, delivered in a closed induction chamber until loss of reflexes was confirmed. After deep anesthesia, the pups were euthanized through rapid decapitation with scissors, following institutional animal care guidelines and international recommendations (CONCEA/AVMA). Brains were immediately removed, mechanically dissociated, and the tissue fragments placed in culture flasks containing DMEM/F12 supplemented with 10% fetal bovine serum (FBS) and antibiotics, maintained at 37 °C in a humidified incubator with 5% CO_2_. Mixed glial cultures were maintained for 14 days to allow cell expansion. To separate astrocytes and microglia, the flasks were subjected to orbital shaking at 200 rpm overnight at 37 °C. Detached microglia were collected from the supernatant, while adherent astrocytes remained attached to the flask. After separation, cells were counted and plated at the desired density for subsequent experiments.

### 2.3 Virus

All the experiments using SARS‐CoV‐2 were performed in a Biological Safety Level 3 (BSL3) laboratory, in accordance with the World Health Organization (WHO) recommendations. The SARS-CoV-2 Gamma strain (P1, GenBank: MZ264787) was titrated by tissue culture infectious dose (TCID_50_) method as described previously (32), and were used for viral infections.

### 2.4 Tissue culture infectious dose assay

Vero E6 cells were seeded in 96-well tissue culture plates (Corning, 3340) at a density of 1×10^4^ cells per well. The next day, virus samples were 10-fold diluted in 100 μL of minimal essential medium (MEM, Gibco) supplemented with 2% FBS and used to infect the cells for 1 hour, at 37 °C in a 5% CO_2_ incubator. The inoculum was removed, replaced with 100 μL of 1× MEM containing 2% FBS, and the cells were incubated at 37 °C in a 5% CO_2_ incubator for 72 hours. The wells were then evaluated microscopically for the presence of characteristic SARS‐CoV‐2 cytopathic effects (CPEs), and TCID_50_ titers were calculated using the Reed–Muench method (33).

### 2.5 Treatment with agonists and inhibitors

To induce the transcription of inflammasome components, cells were treated with LPS (200 ng/mL) (InvivoGen, San Diego, CA, USA) for 3 h. Nigericin (10 nM/mL, 1 h) (InvivoGen) was added directly to the cultures. When pertinent, cells were treated with cathepsins, K^+^ efflux, and ROS pharmacological inhibitors (25 mM CA-074Me [Sigma-Aldrich, 205531], 30 mM KCl [Sigma-Aldrich, P4504], and 200 mM Apocynin [Sigma-Aldrich, 178385], respectively) for 1 h before the stimuli and maintained during the entire experiment.

### 2.6 Treatment with recombinant SARS-CoV-2 proteins

Recombinant SARS-CoV-2 nucleocapsid (N) or spike (S) proteins were delivered intracellularly using Lipofectamine 3000 (Thermo Fisher Scientific), according to the manufacturer’s instructions. Briefly, recombinant proteins were complexed with Lipofectamine 3000 in Opti-MEM reduced-serum medium and added to the cells. Control groups received the transfection reagent alone. After treatment, cells were maintained under standard culture conditions for the indicated experimental time points prior to downstream analyses.

### 2.7 Glial cell infection

Astrocytes from mice were plated in 48-well plates (Costar) at a concentration of 3 × 10^5^ cells per well (for quantitative real-time PCR) or 3 × 10^4^ cells per well in a 96-well plate (Costar) (for immunofluorescence). Then, the cells were infected with SARS-CoV-2 at MOIs of 0.1 and 1 for 2 hours in DMEM/F12 medium without fetal bovine serum (FBS). After this period, the plate was washed to remove extracellular viruses and incubated at 37 °C for 24, 48, and 72 h for subsequent analysis. All experiments were conducted in the Biosafety Level 3 Laboratory at UNIFESP.

### 2.8 Molecular determination of the viral load

To determine the viral load of the infected cells, RT-qPCR was performed. Astrocytes were incubated and treated as described above. Total cell RNA was isolated using the TRIzol method (Thermo Fisher Scientific, 15596026). To purify viral RNA from the supernatants of the infected cultures, the QIAamp Viral RNA Mini Kit (Qiagen, 52906) was used, following the manufacturer’s instructions. The concentration and purity of the mRNA were analyzed by a NanoDrop 2000c spectrophotometer (Thermo Fisher Scientific, Inc.). The absorbance of the samples was evaluated at 260/280 and 260/230 nm, where only ratios > 1.8 were used, indicating the absence of contaminants. cDNA was generated from 500 ng of total RNA using a High-Capacity cDNA Reverse Transcription Kit (catalog number: 4368814) according to the manufacturer’s instructions. cDNA was homogenized with TaqMan Universal PCR Master Mix (Applied Biosystems, 4369016). SARS-CoV-2 viral loads were detected using viral Rdrp expression (34). The Rdrp primers and probe sequences were as follows: forward, 5’- CACATTGGCACCCGCAATC-3’; reverse, 5’- GAGGAACGAGAAGAGGCTTG-3’; and the TaqMan FAM probe, FAM-ACTTCCTCAAGGAACAACATTGCCA-BBQ. Reactions were conducted in a 7500 Real-Time PCR system (Applied Biosystems). Cycle threshold values ​​(Ct) were converted to PFU/mL using a quantitative RNA curve constructed with each point of the serial dilution of viral stock used to determine the PFU/mL titer (34).

### 2.9 Measurement of cytokines

IL-1β cytokine quantification was performed by collecting the supernatant, and a sandwich ELISA was performed according to the manufacturer’s instructions (Invitrogen). The absorbance of the plate was read at 450 nm on a 165 SpectraMax instrument.

### 2.10 Detection of caspase-1 (p10 and p20 domains)

Mature caspase-1 in mice was quantified by collecting the supernatant, and a sandwich ELISA was performed according to the manufacturer’s instructions (AdipoGen Life Sciences). The absorbance of the plate was read at 450 nm on a 165 SpectraMax instrument.

### 2.11 Immunofluorescence

Astrocytes were plated for 72 hours in a 96-well black plate (Greiner) with a clear bottom for microscopy at a density of 3 × 10^4^ per well. After infection (as described above), the supernatant was removed, and the cells were fixed with 3% paraformaldehyde (Sigma-Aldrich) diluted in PBS for at least 30 minutes. Then, the wells were washed twice with warm PBS, followed by blocking/permeabilization buffer [10% BSA (Sigma Aldrich), 1% FBS (LGC), 0.5% Triton-X 100 (Sigma Aldrich), diluted in PBS] for 30 min at room temperature. The wells were carefully washed twice with warm PBS and incubated overnight at 4 °C with 1:2000 anti-GFAP (ab4674), 1:1000 anti-ASC (04-147 - MERCK), and 1:750 anti-spike antibodies. The next day, the wells were rewashed with warm PBS and incubated with Alexa Fluor 488 (Abcam), Alexa Fluor 555 (Abcam), or Alexa Fluor 647 (Invitrogen) secondary antibodies (1:1000) for 1 h at room temperature. The wells were rewashed and incubated with 5 μg/mL DAPI (blue) (Sigma-Aldrich), and images were acquired using an IN Cell 200 Analyzer 2200 instrument.

### 2.12 Cell lysis analysis

Astrocyte lysis was evaluated after cell treatments, as described above, using a lactate dehydrogenase (LDH) activity assay kit according to the manufacturer’s instructions (Sigma–Aldrich).

### 2.13 Statistical analysis

Statistical analyses were performed using GraphPad Prism 9.3.0 (GraphPad Software, San Diego, CA, USA). For each condition, three independent biological experiments were performed, each with three technical replicates. Each data point represents the mean of three technical replicates from one biological replicate (n = 3). Two-way ANOVA or One-way ANOVA was applied to the datasets, followed by the appropriate *post hoc* tests. Data are expressed as mean ± SD from three independent experiments.

## 3 Results

### 3.1 SARS-CoV-2 infects and induces inflammasome activation in astrocytes

Initially, we investigated whether SARS-CoV-2 could infect and activate inflammasomes in astrocytes. To this end, primary cortical astrocytes obtained from newborn mice (E0-E3) were pre-treated with LPS to induce the transcriptional priming of inflammasome components, such as NLRP3 and pro–IL-1β, a step known to be required for full activation of canonical inflammasome pathways (35). Subsequently, astrocytes were infected *in vitro* with the P.1 (Gamma) strain of SARS-CoV-2 at multiplicities of infection (MOI) of 0.1 and 1.

**Figure 1:**
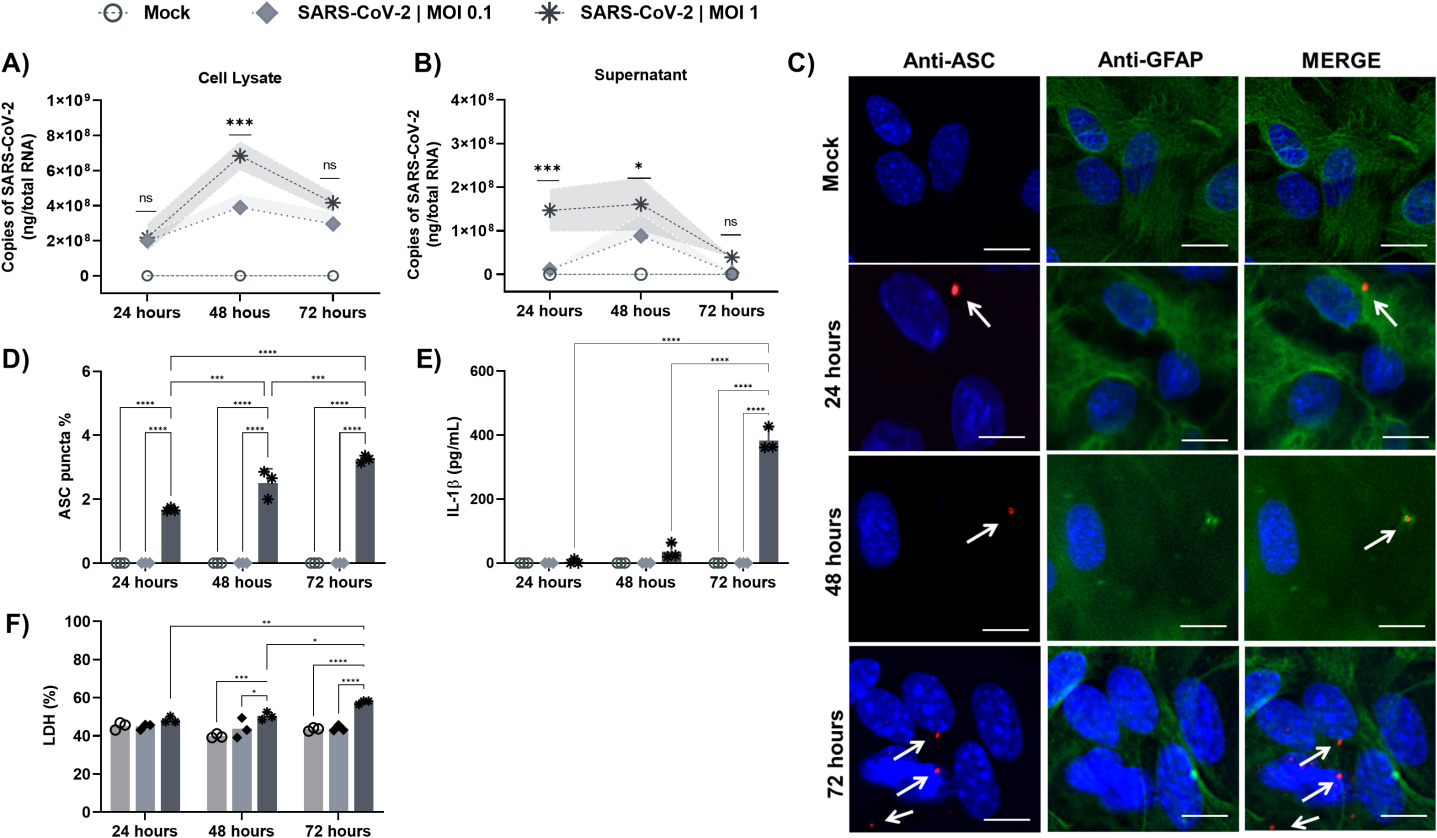
SARS-CoV-2 Infects and induces inflammasome activation in astrocytes. Astrocytes derived from wild-type (WT) mice primed with LPS (200 ng/mL) for 3 h were infected with SARS-CoV-2 at MOIs of 0.1 and 1. RNA was extracted at 24, 48, and 72 h postinfection to assess the SARS-CoV-2 titer by RT–qPCR. **(A)** Viral load in WT astrocytes in cell lysate **(B)** Viral load in WT astrocytes in supernatant. **(C)** Representative immunofluorescence images showing GFAP^+^ astrocytes (green), ASC (red), and nuclei stained with DAPI (blue). White arrows indicate ASC specks, scale bars = 50 μm. **(D)** Quantification of ASC speck formation; **(E)** Measurement of IL-1β release in the supernatant by ELISA. **(F)** Cell viability was verified by LDH release in the supernatant. Statistical analysis was performed using 2way ANOVA: *p < 0.05; **p < 0.01; ***p < 0.001, ****p < 0.0001; ns = not significant). Each data point represents the average of technical replicates from one independent experiment (*n* = 3). Bars represent the mean ± SD across independent experiments.

SARS-CoV-2 infected and replicated in astrocytes in a MOI-dependent manner, as higher viral load was obtained in cell lysates (**Figure 1A**) and supernatants (**Figure 1B**) from MOI of 1-infected cells. Viral replication peaked at 48 hours post-infection, particularly in cell lysates (**Figure 1A**), and progressively declined from 72 hours, culminating in complete clearance from the supernatants (**Figure 1B**), suggesting that astrocytes are capable of controlling the infection over time.

Although both MOIs (0.1 and 1) enabled viral infection replication, only the MOI of 1 induced classical inflammasome activation markers, such as the formation of ASC specks (**Figures 1C, D**), the release of IL-1β (**Figure 1E**), and the release of lactate dehydrogenase (LDH), used as a marker of lytic cell death (**Figure 1F**). These activation signals were more prominent 72 hours post-infection.

Importantly, SARS-CoV-2 also replicated (**Supplementary Figures 1A, B**) and induced ASC specks formation (**Supplementary Figures 1C, D**), caspase-1 activation (**Supplementary Figure 1E**) and IL-1β release (**Supplementary Figure 1F**) in astrocytes that were not pre-treated with LPS, indicating that SARS-CoV-2 is capable of triggering inflammasome activation in both primed and unprimed astrocytes, although potentially with different magnitudes.

### 3.2 SARS-CoV-2 induces NLRP3 inflammasome activation in astrocytes

**Figure 2:**
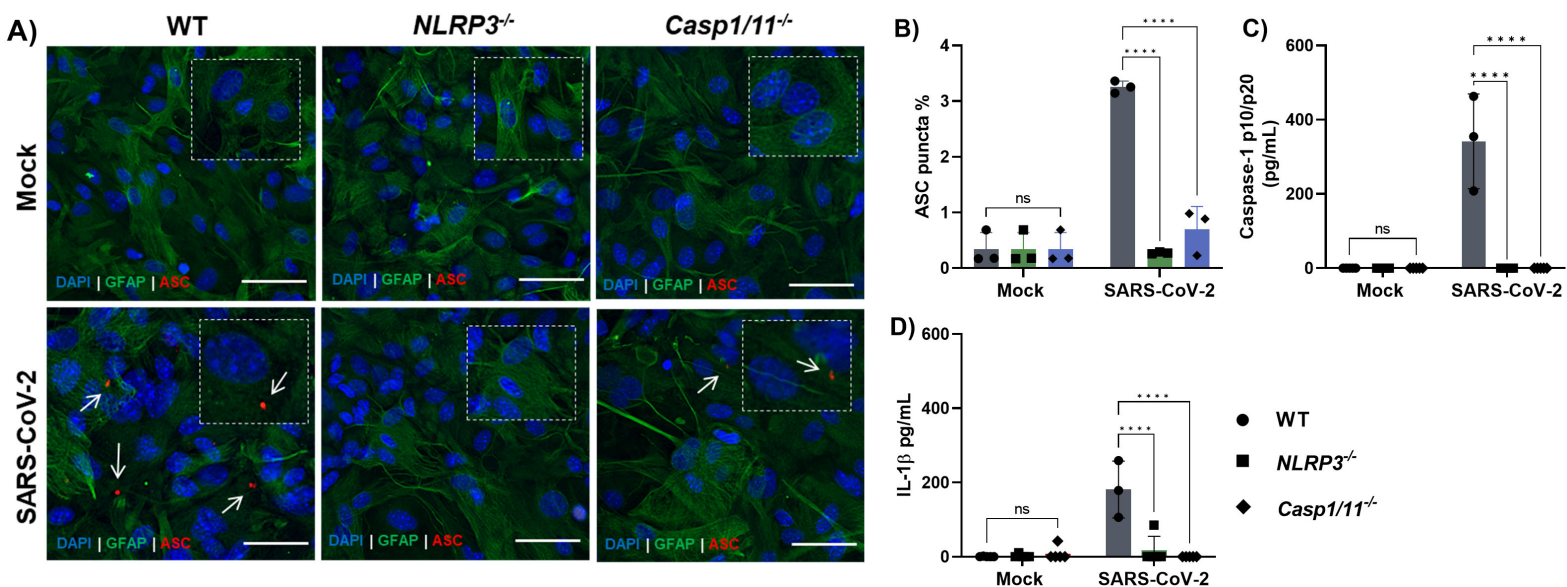
SARS-CoV-2 infection induces NLRP3 inflammasome activation in astrocytes. Astrocytes derived from WT, *NLRP3^-/-,^* and *caspase-1/11^-/-^* mice primed with LPS (200 ng/mL) for 3 h were infected with SARS-CoV-2 at an MOI of 1 for 72 h. **(A)** Representative immunofluorescence images showing GFAP^+^ astrocytes (green), ASC (red), and nuclei stained with DAPI (blue). White arrows indicate ASC specks, scale bars = 50 μm. **(B)** Quantification of ASC speck formation; **(C)** Measurement of caspase-1 and **(D)** IL-1β release in the supernatant by ELISA. Statistical analysis was performed using 2way ANOVA: ****p < 0.0001; ns = not significant. Each data point represents the average of technical replicates from one independent experiment (*n* = 3). Bars represent the mean ± SD across three independent experiments.

Given that NLRP3 inflammasome activation by SARS-CoV-2 has been extensively documented in both human and murine monocytes (21–24, 26, 36). We investigated whether a similar mechanism occurs in astrocytes. To this end, astrocytes derived from NLRP3-deficient (*Nlrp3*^^-^/^-^^) and caspase-1/11-deficient (*Casp1/11*^^-^/^-^^) mice were infected with SARS-CoV-2 (MOI of 1). In both knockout models, ASC speck formation (**Figures 2A, B**), caspase-1 activation (**Figure 2C**), and IL-1β secretion (**Figure 2D**) were significantly reduced compared to wild-type controls.

Importantly, similar reductions were observed in astrocytes that were not pre-treated with LPS (**Supplementary Figures 2A-D**), further supporting the conclusion that SARS-CoV-2 activates the NLRP3 inflammasome pathway in astrocytes under both primed and unprimed conditions.

### 3.3 SARS-CoV-2 N- and S- proteins activate NLRP3 through common cytosolic pathways

**Figure 3:**
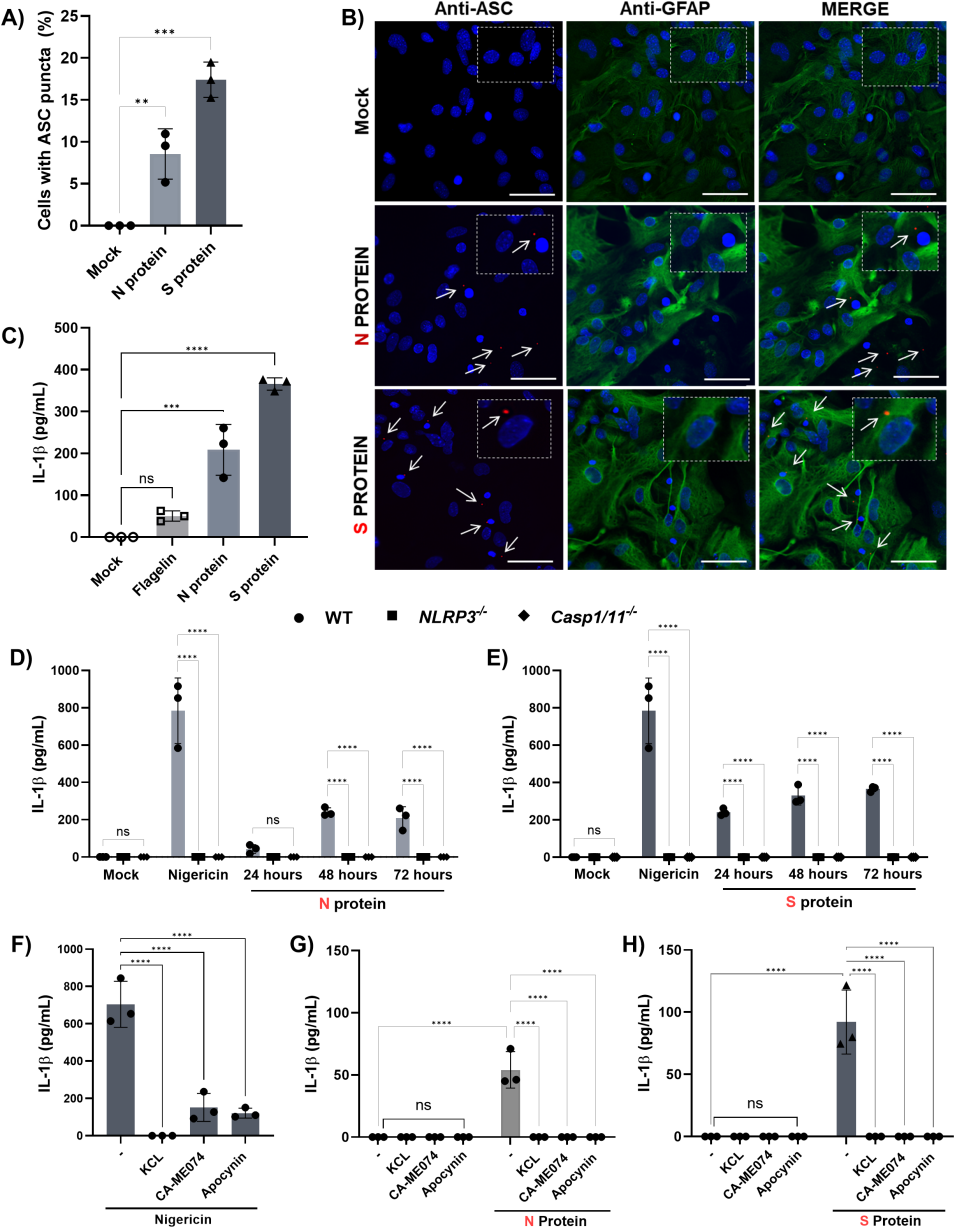
NLRP3 activation by SARS-CoV-2 in astrocytes depends on the viral N and S proteins and involves common cytosolic pathways. Astrocytes derived from WT mice primed with LPS (200 ng/mL) for 3 h were treated with 100 ng of recombinant N or S proteins of SARS-CoV-2 delivered using Lipofectamine 3000. Flagellin (6 µg/mL) was used as an unrelated protein control for 72 h. **(A)** Quantification of ASC speck formation; **(B)** Representative immunofluorescence images showing GFAP+ astrocytes (green), ASC (red), and nuclei stained with DAPI (blue). White arrows indicate ASC specks, scale bars = 50 μm. **(C)** Measurement of IL-1β by ELISA. **(D–H)** Astrocytes derived from WT, *NLRP3^-/-,^* and *caspase-1/11^-/-^* mice primed with LPS (200 ng/mL) for 3 h were treated with 100 ng of recombinant N or S proteins of SARS-CoV-2 using lipofectamine 3000 for 72 h, or with nigericin (10 nM/mL, 1 hour). **(D)** Measurement of IL-1β by ELISA in response to N protein and **(E)** S protein. **(F)** WT astrocytes treated with Nigericin (10 nM/mL, 1 hour) in the presence of inhibitors of potassium efflux (KCl - 30 mM), lysosomal cathepsin B (CA074-Me - 25 µM), or mitochondrial ROS (apocynin - 200 µM). **(G)** WT astrocytes treated with N or **(H)** S proteins in the presence of inhibitors of potassium efflux (KCl - 30 mM), lysosomal cathepsin B (CA074-Me - 25 µM), or mitochondrial ROS (apocynin - 200 µM). Statistical analysis was performed using **(A, B, F–H)** one-way ANOVA: **p < 0.01; ***p < 0.001, ****p < 0.0001; ns = not significant) and (D and E) 2way ANOVA: ****p < 0.0001; ns = not significant. Each data point represents the average of technical replicates from one independent experiment (*n* = 3). Bars represent the mean ± SD across three independent experiments.

To investigate the mechanisms by which SARS-CoV-2 induces NLRP3 activation in astrocytes, we stimulated these cells with recombinant nucleocapsid (N) and spike (S) viral proteins after LPS priming. Both proteins induced ASC speck formation (**Figures 3A, B**) and IL-1β secretion by astrocytes, in contrast to flagellin, an NLRC4 agonist, or an unrelated control protein (**Figure 3C**). Notably, astrocyte responses to N (**Figure 3D**) and S (**Figure 3E**) proteins were significantly reduced in the absence of NLRP3 or caspase-1, indicating that SARS-CoV-2 triggers NLRP3 activation via its structural proteins N and S.

Since NLRP3 activation in immune cells is typically triggered by cytosolic disturbances induced by PAMPs or DAMPs, such as K^+^ efflux (37), cathepsin release (38), and mitochondrial ROS generation (39), we investigated whether these signals were required for NLRP3 activation in response to SARS-CoV-2 in astrocytes, similar to the classic agonist nigericin (**Figure 3F**). We observed that the inhibition of these pathways abolished IL-1β production in response to the N (**Figure 3G**) and S (**Figure 3H**) proteins, confirming that SARS-CoV-2 activates NLRP3 in astrocytes through classical signaling pathways triggered by its viral proteins.

### 3.4 NLRP3 inflammasome restricts SARS-Cov-2 replication in astrocytes

**Figure 4:**
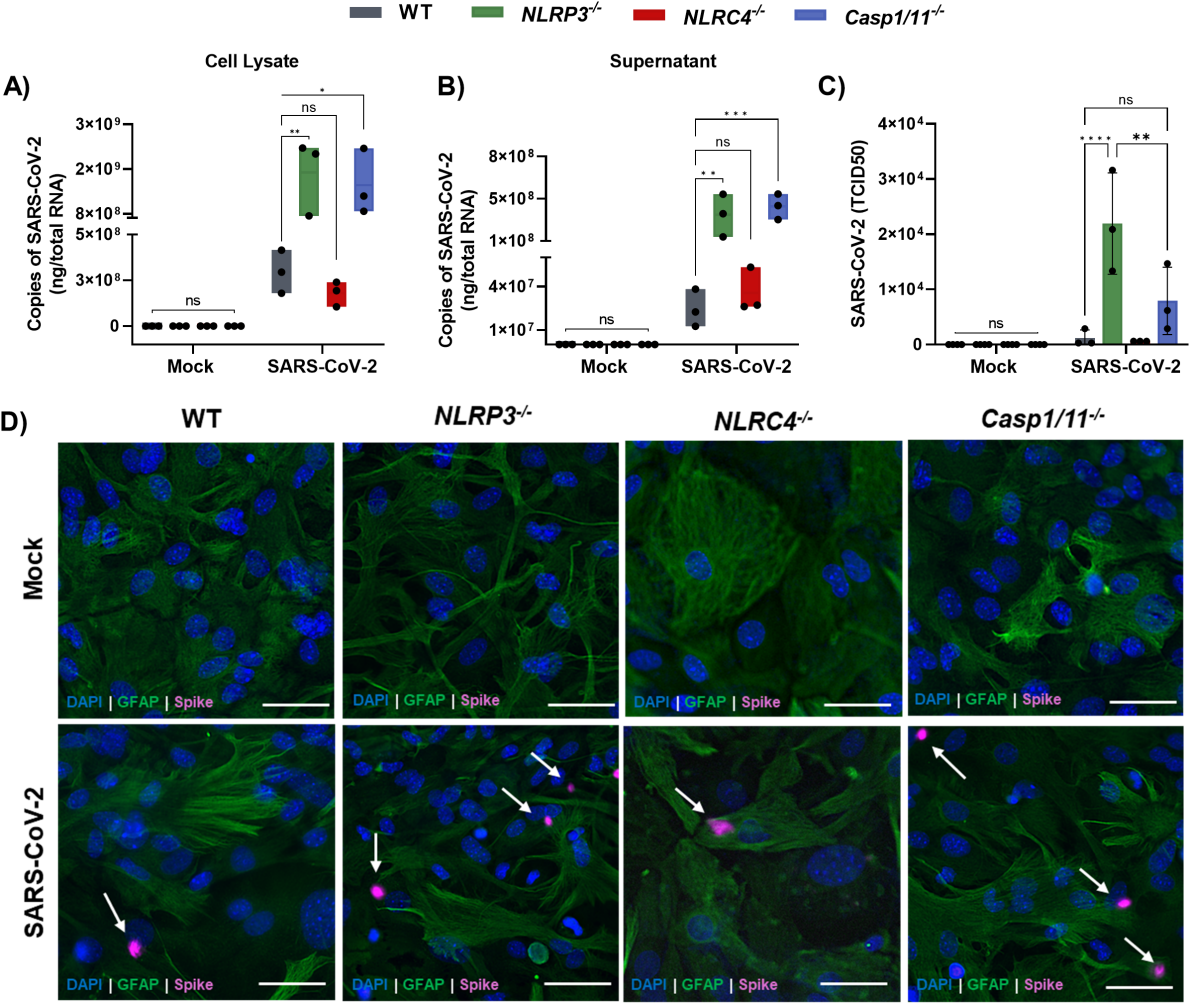
NLRP3, but not NLRC4, is required for the control of SARS-CoV-2 replication in astrocytes. Astrocytes derived from WT, *NLRP3^-/-^*, *NLRC4^-/-,^* and *caspase-1/11^-/-^* mice primed with LPS (200 ng/mL) for 3 h were infected with SARS-CoV-2 at an MOI of 1 for 72 h. **(A)** Viral load in WT astrocytes in cell lysate **(B)** Viral load in WT astrocytes in supernatant. **(C)** Infectious Dose 50% Tissue Culture Infectivity Dose (TCID50) assay from uninfected and infected astrocytes derived from WT, *NLRP3^-/-^*, *NLRC4^-/-,^* and *caspase-1/11^-/-^* mice. **(D)** Representative images of cells showing the presence of spike protein in the cells. White arrows indicate spike proteins. Statistical analysis was performed using 2way ANOVA: *p < 0.05; **p < 0.01; ***p < 0.001; ****p < 0.0001; ns = not significant). Each data point represents the average of three technical replicates from an independent biological experiment (*n* = 3). Bars represent the mean ± SD across three independent experiments.

To evaluate the role of the NLRP3 inflammasome in controlling SARS-CoV-2, we quantified the viral load in astrocytes deficient in components of this inflammasome. A significantly higher viral load was observed in *Nlrp3^-/-^* and *casp-1/11^-/-^* astrocytes 72 h post-infection, but not in *Nlrc4^-/-^* cells, both in cell lysates (**Figures 4A**) and in supernatants (**Figure 4B**), consistent with the virus staining inside cells (**Figure 4D**). Notably, again, similar results were observed in the absence of LPS priming (**Supplementary Figures 2E, F**). Moreover, the release of infectious viral particles, as determined by TCID_50_ assay, was markedly increased in the absence of NLRP3 (**Figure 4C**), further confirming its role in antiviral defense. Collectively, these results demonstrate that the NLRP3 inflammasome plays a crucial role in limiting SARS-CoV-2 replication in astrocytes.

### 3.5 Gasdermin-D-dependent IL-1β release is involved in the control of SARS-CoV-2 by astrocytes

**Figure 5:**
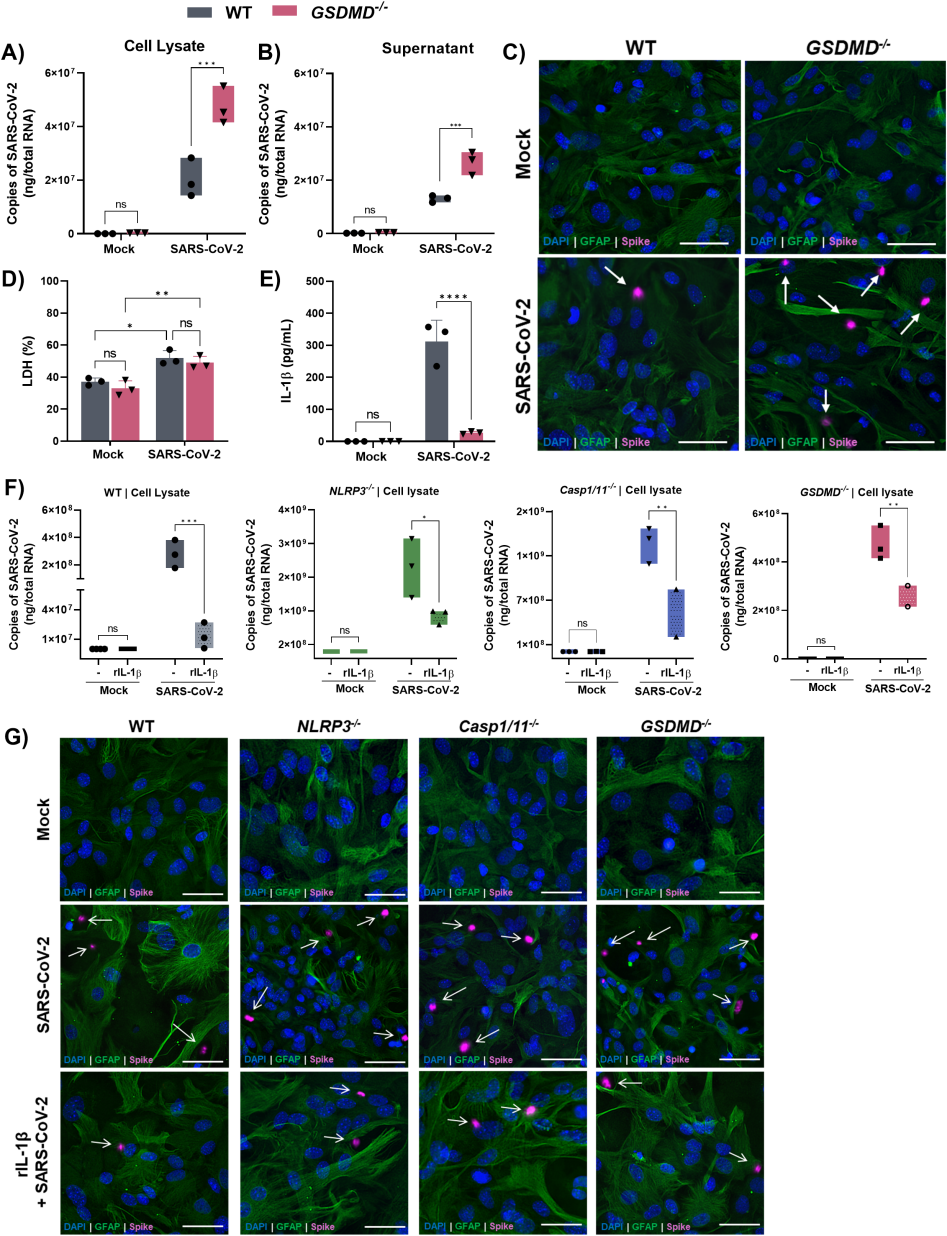
Gasdermin-D promotes the control of SARS-CoV-2 infection in astrocytes. Astrocytes derived from WT and *GSDMD^-/-^* mice primed with LPS (200 ng/mL for 3 h) were infected with SARS-CoV-2 at an MOI of 1 for 72 h. **(A)** Viral load in cell lysate and **(B)** supernatant. **(C)** Representative images of cells showing the presence of spike protein in the cells. White arrows indicate spike proteins. **(D)** Cell viability was verified by LDH release in the supernatant. **(E)** Measurement of IL-1β release in the supernatant by ELISA. Astrocytes derived from WT, *NLRP3^-/-^*, *caspase-1^-/-,^* and *GSDMD^-/-^* mice primed with LPS (200 ng/mL) for 3 h were treated or not with recombinant IL-1β (rIL-1β - 20 µM) and infected with SARS-CoV-2 at an MOI of 1 for 72 h **(F)**. Viral load in WT astrocytes in cell lysate. **(G)** Representative images of cells showing the presence of spike protein in the cells. White arrows indicate spike proteins. Statistical analysis was performed using 2way ANOVA: *p < 0.05; **p < 0.01; ***p < 0.001, ****p < 0.0001; ns = not significant). Each data point represents the average of three technical replicates from an independent biological experiment (*n* = 3). Bars represent the mean ± SD across three independent experiments.

After establishing the role of the NLRP3 inflammasome in controlling SARS-CoV-2 replication, we next investigated the downstream effector mechanisms involved. Inflammasome activation typically triggers pyroptosis and cytokine release via GSDMD pore formation (40–42). To assess the contribution of GSDMD, we infected astrocytes from *Gsdmd*-deficient mice. Compared to wild-type (WT) controls, *Gsdmd*^^-^/^-^^ astrocytes exhibited significantly higher viral loads in both cell lysates and supernatants (**Figures 5A–C**). However, no differences in LDH release were detected between WT and *Gsdmd*^^-^/^-^^ astrocytes (**Figure 5D**), indicating that GSDMD-mediated pyroptosis is not the primary mechanism of infection control. Instead, IL-1β levels were markedly reduced in *Gsdmd*^^-^/^-^^ cultures (**Figure 5E**), suggesting that GSDMD regulates viral control through IL-1β release. Supporting this, the addition of exogenous IL-1β led to a dose-dependent reduction in viral load (**Supplementary Figures 3A, B**). Importantly, viral control strongly correlated with IL-1β levels across cultures (**Supplementary Figure 3C**), and exogenous IL-1β did not affect astrocyte viability, as assessed by LDH release (**Supplementary Figure 3D**).

Furthermore, recombinant IL-1β restored antiviral activity in *Nlrp3*^^-^/^-^^, *Casp1/11*^^-^/^-^^, and *Gsdmd*^^-^/^-^^ astrocytes, as shown by reduced intracellular viral staining (**Figure 5G**) and decreased viral RNA (**Figure 5F**). These data indicate that IL-1β release downstream of GSDMD is a key effector mechanism for limiting SARS-CoV-2 in astrocytes. Importantly, even without LPS, the absence of GSDMD still resulted in increased viral load, and IL-1β supplementation was sufficient to restore antiviral responses (**Supplementary Figure 4**). Together, these findings highlight that astrocytes possess an intrinsic, inflammasome-dependent mechanism to restrict SARS-CoV-2 replication via GSDMD-mediated IL-1β release, independent of exogenous priming signals.

## 4 Discussion

Inflammasomes play a crucial role in controlling infections mediated by innate immune cells (43). Recent studies have unveiled their ability to activate effector mechanisms in non-immune cells as well (44), including intestinal epithelial cells (IECs) (45, 46), endothelial cells (47), renal epithelial cells (48), keratinocytes (49), osteoclasts (50), and cells of the central nervous system (CNS) (51). In astrocytes, inflammasome activation can result in beneficial or detrimental consequences, depending on the context (52–58). However, the role of inflammasomes in the autonomous ability of astrocytes to control infections remains poorly investigated. A recent report from our group demonstrated that caspase-1/11 plays a non-conventional role in the astrocyte’s ability to control ZIKV infection (59). Even in the absence of classical signals of inflammasome activation (e.g., ASC specks, IL-1β, pyroptosis), caspase-1 activation by ZIKV seems to inhibit the glycolytic pathway, thus preventing ZIKV replication within astrocytes. Here, we demonstrate that SARS-CoV-2 triggers canonical NLRP3 inflammasome activation in murine astrocytes, resulting in GSDMD-dependent IL-1β secretion, which plays a crucial role in controlling viral replication.

Notably, we show that hallmark features of inflammasome activation (e.g., ASC specks, IL-1β, and caspase-1 cleavage) occur in response to SARS-CoV-2 infection even in the absence of LPS priming, indicating that astrocytes possess intrinsic pathogen-sensing mechanisms capable of initiating inflammasome assembly upon viral challenge. The exacerbated viral replication observed in *Nlrp3^^-^/^-^^* and *caspase-1/11^^-^/^-^^* astrocytes under unprimed conditions further underscores the functional relevance of this pathway. Together, these findings emphasize the physiological importance of astrocytic inflammasome signaling as an essential early defense mechanism against neurotropic viral infections.

While much research has focused on the SARS-CoV-2 impact on the respiratory system, its effects on the CNS have garnered significant attention due to the diverse neurological symptoms observed in infected individuals. These include cognitive impairments (60, 61), encephalopathy (62–64), and stroke (65–67), suggesting that SARS-CoV-2 may influence neuroinflammatory processes within the CNS. However, it remains to be elucidated whether neuroinflammation results from the presence of the virus, the leakage of inflammatory mediators crossing the blood-brain barrier, or both. In fact, SARS-CoV-2 variants of concern (VOCs), such as the Wuhan, beta, and delta variants, seem to infect neural cells, including astrocytes (10, 68, 69). Although neurotropism has not yet been demonstrated for the Gamma variant, its phylogenetic proximity to its predecessors and the presence of shared mutations, such as D614G, support our findings that SARS-CoV-2 Gamma infects and replicates in astrocytes. This is confirmed by viral load measurements and the detection of viral proteins within these cells, demonstrating that astrocytes support the full viral life cycle and release infectious virions, as observed in the TCID_50_ assay. However, the activation of inflammasomes was observed only at an MOI of 1, thus indicating that a low viral load is insufficient to induce the assembly of these platforms. Importantly, inflammasome activation is required for rapid virus clearance, as astrocytes from WT mice can clear the virus within 72 hours after infection. These findings are consistent with previous studies highlighting the critical role of the NLRP3 inflammasome in the immune response to viral infections, including SARS-CoV-2 (17, 21–23).

GU-rich genomic RNA (21), ORF3a viroporin (23), and the N protein (24, 25) have been identified as activators of the NLRP3 inflammasome in THP1 and A549 cell lines. Interestingly, while the N protein induces inflammasome activation, it has also been reported to inhibit GSDMD, thereby blocking pyroptosis and the release of IL-1β. Conversely, the S protein upregulates NLRP3 expression and induces IL-1β release in macrophages from COVID-19 patients (70). In astrocytes, N- and S proteins from SARS-CoV-2 activated NLRP3 by a pathway that involves K^+^ efflux, ROS generation, and lysosomal cathepsin release, as described for monocytes (22). Furthermore, our study highlights the importance of downstream inflammasome mechanisms in controlling viral infection. Although GSDMD is known as the key effector of pyroptosis (39–41), *Gsdmd*-deficient astrocytes exhibited increased SARS-CoV-2 replication without a corresponding increase in cell death. This indicates that pyroptosis is not the primary mechanism by which the inflammasome restricts SARS-CoV-2 replication in astrocytes, contrasting with findings in monocytes (71). Instead, GSDMD appears to play a critical role in IL-1β release, underscoring the importance of cytokine-mediated antiviral defense over cell death mechanisms.

Accordingly, the recombinant IL-1β treatment significantly reduced viral load and infectious particles in both wild-type and inflammasome-deficient astrocytes. Interestingly, while IL-1β effectively suppressed viral replication, it did not induce cell death, suggesting that IL-1β limits viral replication without compromising astrocyte viability. Although the precise mechanism by which IL-1β suppresses SARS-CoV-2 replication in astrocytes remains to be elucidated, it is known that astrocyte-secreted IL-1β can act through both paracrine and autocrine signaling (66, 67), potentially affecting nearby cells, such as neurons and microglia.

Collectively, our findings reveal that SARS-CoV-2 can trigger inflammasome activation in astrocytes under both primed and unprimed conditions. While LPS priming remains a valuable experimental tool for amplifying and dissecting inflammasome-related responses, activation under unprimed conditions likely provides a more physiologically relevant representation of innate immune mechanisms operating in the CNS during viral infection. As the most abundant cell type in the CNS and key sites for SARS-CoV-2 replication (10–14), astrocytes play a crucial role in viral control. Understanding the involvement of inflammasomes in their autonomous ability to restrict virus replication is therefore of fundamental importance. While the potentially harmful effects of neuroinflammation must be carefully managed, targeting the inflammasome presents a promising strategy to limit SARS-CoV-2 spread in brain cells.

## Data Availability

The original contributions presented in the study are included in the article/**Supplementary Material**. Further inquiries can be directed to the corresponding author.

## Glossary

A549: Human lung carcinoma cell line
ACE2: Angiotensin-converting enzyme 2
AIM2: Absent in melanoma 2
ASC: Apoptosis-associated speck-like protein containing a CARD
CA-074Me: Cathepsin B inhibitor CA-074 methyl ester
CARD: Caspase activation and recruitment domain
Casp1/11: Caspase-1/11
cDNA: Complementary DNA
CNS: Central nervous system
CPE: Cytopathic effect
DAMPs: Damage-associated molecular patterns, DAPI, 4′,6-diamidino-2-phenylindole
DMEM/F12: Dulbecco’s Modified Eagle Medium/Nutrient Mixture F-12
ELISA: Enzyme-linked immunosorbent assay
GFAP: Glial fibrillary acidic protein
GSDMD: Gasdermin-D
IEC: Intestinal epithelial cell
IL-1β: Interleukin-1 beta
KCl: Potassium chloride
LDH: Lactate dehydrogenase
LPS: Lipopolysaccharide
MOI: Multiplicity of infection
NLRP3: NOD-like receptor family pyrin domain containing 3
NLRC4: NLR family CARD domain-containing 4
ORF3a: Open reading frame 3a
PAMPs: Pathogen-associated molecular patterns
PFU: Plaque-forming units
PRRs: Pattern recognition receptors
Rdrp: RNA-dependent RNA polymerase
SARS-CoV-2: Severe acute respiratory syndrome coronavirus 2
TCID50: Tissue culture infectious dose 50
THP1: Human monocytic cell line
VOC: Variant of concern
ZIKV: Zika virus
