# Supplementary material for: NLRP3 inflammasome activation in astrocytes restricts SARS-CoV-2 through gasdermin-D-driven IL-1β release
Source: Front Immunol. 2026 Jan 20;16:1703765. doi: 10.3389/fimmu.2025.1703765 (PMC12864467; doi:10.3389/fimmu.2025.1703765)
Supplement: Supplementary file 1 [file Supplementaryfile1.docx]

**Supplementary Material**

1. **Supplementary Figures**

**
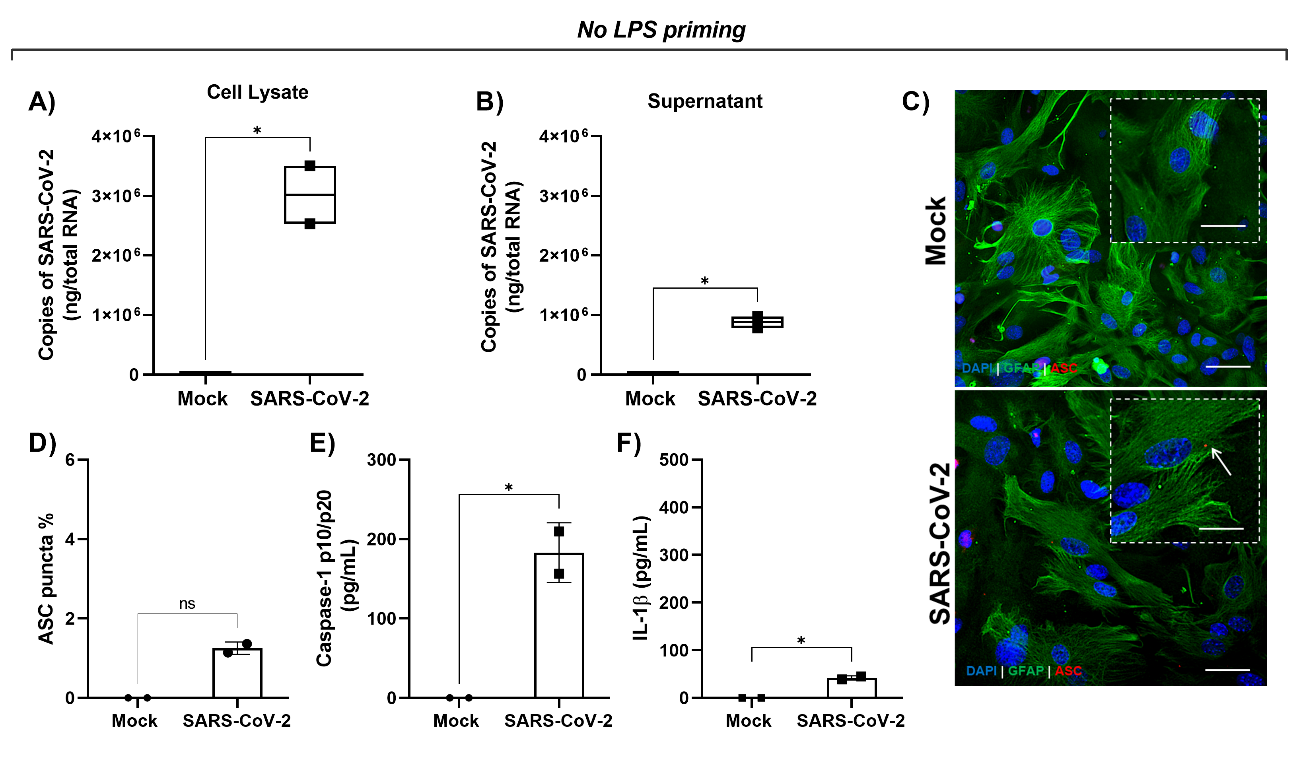
Supplementary Figure 1. SARS-CoV-2 activates the inflammasome pathway in murine astrocytes in the absence of LPS priming.**Astrocytes derived from WT mice were infected with SARS-CoV-2 (MOI of 1) for 72 h. Viral RNA levels in **(A)** cell lysates and **(B)** supernatants were quantified by RT-qPCR. **(C)** Representative immunofluorescence images showing GFAP^+^ astrocytes (green), ASC (red), and nuclei stained with DAPI (blue). White arrows indicate ASC specks, scale bars = 50 μm. **(D)** Quantification of ASC speck formation, **(E)** Caspase-1 p10/p20 levels and **(F)** IL-1β secretion measured by ELISA. Statistical analysis was performed using Welch's t test: ns = not significant; *p < 0.05; Each data point represents the average of technical replicates from one independent experiment (*n* = 2). Bars represent the mean ± SD across two independent experiments.

**
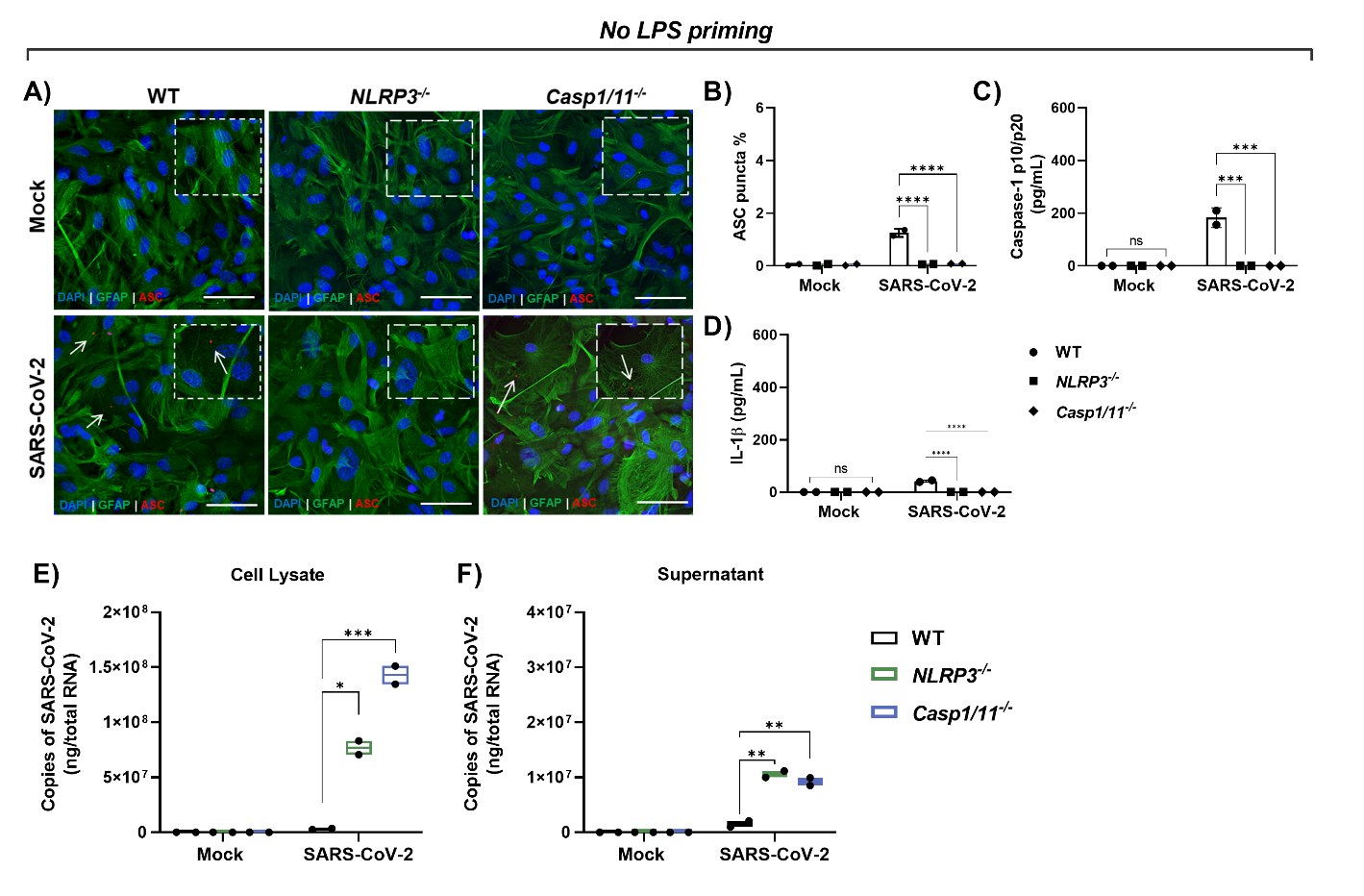
**

**Supplementary Figure 2. NLRP3 inflammasome activation in murine astrocytes upon SARS-CoV-2 infection occurs even in the absence of LPS priming.** Astrocytes derived from WT, *Nlrp3^⁻/⁻^,* and *Casp1/11^⁻/⁻^* mice were infected with SARS-CoV-2 (MOI of 1) for 72 h. **(A)** Representative immunofluorescence images showing GFAP^+^ astrocytes (green), ASC (red), and nuclei stained with DAPI (blue). White arrows indicate ASC specks. Scale bars = 50 μm. **(B)** Quantification of ASC speck formation. **(C)** Caspase-1 p10/p20 protein levels measured by ELISA. **(D)** IL-1β secretion measured by ELISA. **(E)** Viral load in WT astrocytes cell lysate and **(F)** supernatant. Statistical analysis was performed using 2-way ANOVA: * p < 0.05, **p < 0.01; ****p < 0.001;* *****p < 0.0001;* ns = not significant. Each data point represents the average of technical replicates from one independent experiment (*n* = 2). Bars represent the mean ± SD across two independent experiments.


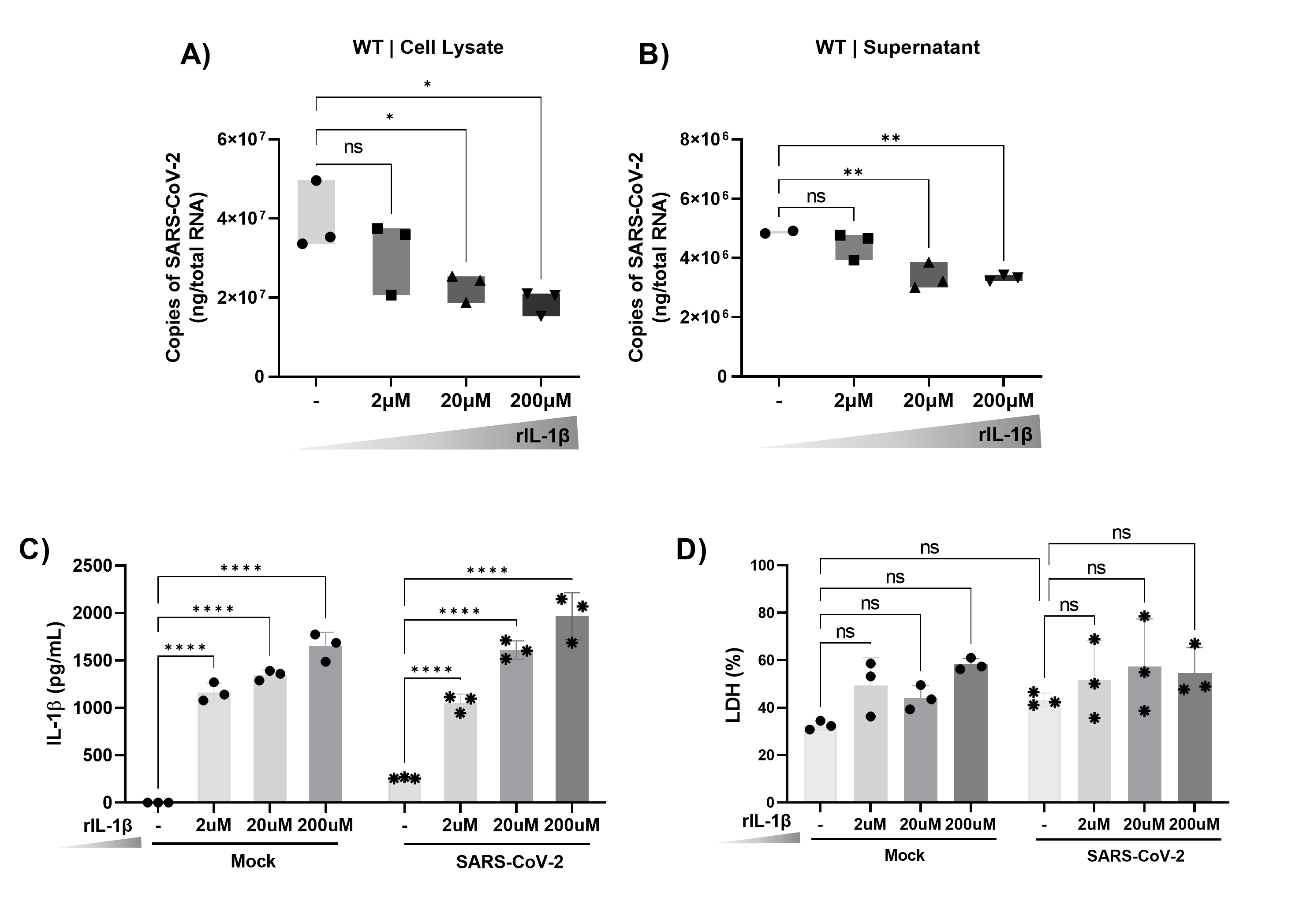


**Supplementary Figure 3. The IL-1β-mediated control of SARS-CoV-2 infection in astrocytes occurs in a dose-dependent manner.** Astrocytes derived from WT mice primed with LPS (200 ng/mL) for 3 h were treated or not with recombinant IL-1β (2, 20 or 200 µM) and infected with SARS-CoV-2 at an MOI of 1 for 72 h. Viral load in WT astrocytes in **(A)** cell lysate and **(B)** supernatant **(C)** Measurement of IL-1β release in the supernatant by ELISA. **(D)** Cell viability by LDH release in the supernatant. Statistical analysis was performed using one-way ANOVA: * p < 0.05, **p < 0.01; ****p < 0.001;* *****p < 0.0001;* ns = not significant; Representative data of two independent experiments performed in technical triplicate.

**
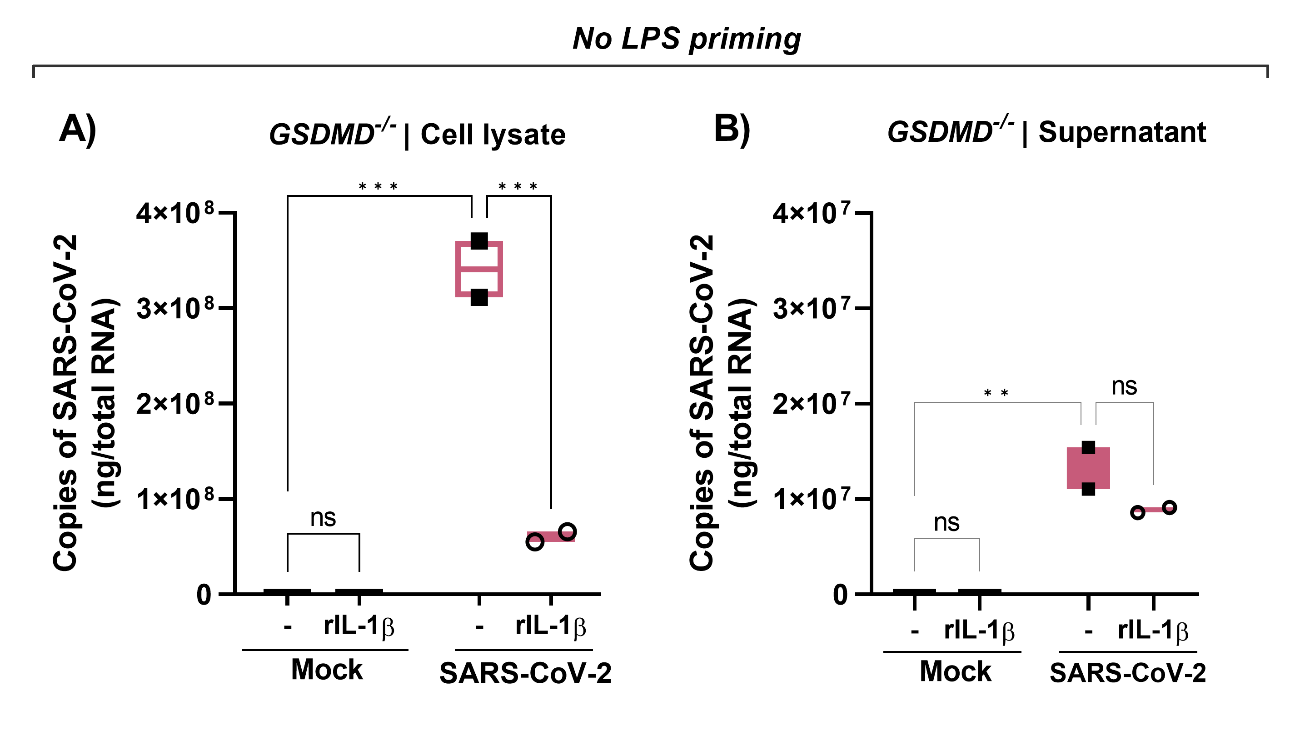
**

**Supplementary Figure 4. GSDMD deficiency increases SARS-CoV-2 replication in astrocytes, and IL-1β supplementation restores antiviral activity in the absence of LPS priming.** Astrocytes derived from WT and *Gsdmd^-/-^* mice were treated or not with recombinant IL-1β (rIL-1β - 20 µM) and infected with SARS-CoV-2 at an MOI of 1 for 72 h. Viral load in **(A)** cell lysate and **(B)** supernatant. Statistical analysis was performed using 2way ANOVA: **p* < 0.05; *****p* < 0.0001; ns = not significant). Each data point represents the average of technical replicates from one independent experiment (*n* = 2). Bars represent the mean ± SD across two independent experiments.
